# The residual cardiorenal risk in type 2 diabetes

**DOI:** 10.1186/s12933-021-01229-2

**Published:** 2021-02-05

**Authors:** Dario Giugliano, Maria Ida Maiorino, Giuseppe Bellastella, Katherine Esposito

**Affiliations:** 1 Division of Endocrinology and Metabolic Diseases, Department of Advanced Medical and Surgical Sciences, University of Campania Luigi Vanvitelli, Naples, Italy; 2 Ph.D. of Translational Medicine, Chair of Endocrinology and Metabolic Diseases, Department of Advanced Medical and Surgical Sciences, University of Campania Luigi Vanvitelli, Naples, Italy; 3 Diabetes Unit, Department of Advanced Medical and Surgical Sciences, University of Campania Luigi Vanvitelli, Naples, Italy

## Abstract

In this commentary, we introduce the concepts of removed and residual risks in conditioning thecardiorenal outlook of patients with type 2 diabetes (T2D). The removed cardiorenal risk represents the risk of progression of CV events (major adverse cardiovascular events, MACE; heart failure, HF) and diabetes kidney disease (DKD) taken away by optimal glycemic control or the use of newer antihyperglycemic drugs (glucagon-like peptide-1 receptor agonists, GLP-1RA, andsodium-glucose transporter-2 inhibitors, SGLT-2i) in patients with T2D, as demonstrated by the results of intensive glucose lowering trials (IGT) and cardiovascular outcome trials (CVOT). IGT have shown that successful glycemic control has modest benefits, as the removed cardiorenal risk ranges from 9% for MACE, to 20% for progression of DKD and to 0% for HF. The removed risk of MACE is 13% for GLP-1RA and 12% for SGLT-2i. However, SGLT-2i, as compared with GLP-1RA, removed twofold more risk (39% vs 17%) for kidney outcomes and fourfold more risk (33% vs 9%) for HF. Dipeptidyl peptidase-4 inhibitors have no clinically important cardiorenal benefits, as residual risk is 99% for MACE, 100% for kidney outcomes (excluding new albuminuria), and 100% for HF. Although the results of some real world, population-based cohort studies suggest the possibility that the cardiorenal protection afforded by newer antihyperglycemic drugs is additive to that of optimal glycemic control, only specific randomized controlled trials could answer this question.

Cardiovascular (CV) disease is the leading cause of morbidity and mortality for people with type 2 diabetes (T2D), resulting from the likely association and contribution of many risk factors, including although not limited to hypertension, dyslipidemia, obesity and diabetes itself. Large benefits are seen when multiple CV risk factors are addressed simultaneously. For instance, patients with T2D who had five risk-factor variables within the target ranges had little [1] or no excess [2] CV risk as compared with the general population, with the notable exception of heart failure (HF) which seems unrelated to major metabolic and CV risk factors. Unfortunately, only 5–6% of people with T2D had optimal risk factor control [1, 2]. Diabetic kidney disease (DKD) occurs in 20–40% of patients with diabetes [3] and is the leading cause of end stage kidney disease (ESKD) in the U.S. Its diagnosis is mainly based on the persistent presence of elevated urinary albumin excretion (albuminuria), low estimated glomerular filtration rate (eGFR), or both. The presence of DKD in T2D has a role in perpetuating the development of adverse CV outcomes [4], although it often does not gain appropriate consideration by clinicians. DKD is an important contributing factor to CV and all-cause mortality, to such an extent that it may be recognized as a coronary heart disease risk equivalent [5]. Accordingly, diabetic vascular complication-related deaths had increased substantially during 2000–2016, mainly driven by the increased mortality of kidney complications [6]. 

Diabetes is defined by a hyperglycemic status: intuitively, glycemic control has long been though fundamental to diabetes management for reducing diabetes complications. Intensive glycemic control (IGC), with the goal of achieving near-normoglycemia, has been shown in large prospective randomized studies (intensive glucose trials, IGT) to delay the onset and progression of DKD in patients with T2D [7]. On the other hand, IGC is thought to have controversial effects in reducing the macrovascular complications associated with T2D. The overall CONTROL (Collaborators on Trials of Glucose Lowering) meta-analysis based on 27,000 patients of the 4 major trials evaluating the effects of IGC on CVD (UKPDS, ACCORD, ADVANCE and VADT) showed that the more-intensive, as compared with the less-intensive glucose control, was associated with a 9% reduction of risk of major adverse cardiovascular events (MACE) and a mean reduction in hemoglobin A1c (HbA1c) level of 0.9% [8]. Moreover, in the subset of diabetic population with pre-existing CVD, the cardiovascular benefit of IGC was completely absent (Hazard Ratio, HR = 1.00, 95% CI, 0.91–1.10) [9]. In a patient-level meta-analysis from the same four IGT, the relative risk for kidney events was reduced by 20% over 5 years of follow-up (HR = 0.80, 95% CI, 0.72 to 0.88) [10]. Although it is difficult to quantify the absolute risk reduction of renal complications because the associated hypoglycemia and polypharmacy may have outweighed their potential benefits, nonetheless lower HbA1c levels were associated with reduced onset or progression of DKD in three IGT. 

The residual cardiorenal risk has been defined as the risk of progression of CV events (MACE or HF) and DKD that remains after the optimal glycemic control in T2D [11]. The first column of Fig. 1 shows the amount of removed risk (green) and residual risk (red) for MACE, DKD and HF in patients with T2D, as demonstrated by the results of IGT: residual risk after successful glycemic control ranges from 91% for MACE, to 80% for progression of DKD, and to 100% for HF. In quantitative terms, progression of DKD ranked first, with 20% removed risk, and hence 80% residual risk. However, the effects of IGC are more evident in terms of reducing albuminuria, as compared with harder renal endpoints such as ESKD, renal death, or fall in eGFR [10]. Despite all the efforts and sacrifices made by patients and their physicians to pursue an optimal glycemic control, it is quite frustrating to acknowledge the limited cardiorenal benefits obtained with IGC in T2D. In the last decade, the use of newer and innovative glucose lowering drugs surprisingly provided a significant reduction in the incidence of CV events, suggesting the possibility of cardiorenal protection beyond glycemic control [12]. Meta-analyses of the cardiovascular outcome trials (CVOT) reported to date suggest that glucagon-like peptide-1 receptor agonists (GLP-1RA) and sodium-glucose transporter-2 inhibitors (SGLT-2i) reduce risk of atherosclerotic MACE to a comparable degree in patients with T2D [13]. As for IGT, the residual cardiorenal risk can be defined as the risk of progression of CV events (MACE or HF) and DKD that remains after treatment with either GLP-1RA or SGLT-2i, as demonstrated by the results of CVOT. Accordingly, the removed risk of MACE is 13% for GLP-1RA and 12% for SGLT-2i (Fig. 1). However, the similarities between the two classes of drugs go no further. Figure 1 also shows that SGLT-2i, as compared with GLP-1RA, removed twofold more risk (39% vs 17%) for kidney outcomes and fourfold more risk (33% vs 9%) for HF. Of note, the renal benefit of GLP-1RA is confined primarily on a new onset of macroalbuminuria [5, 14]. Moreover, it should be stressed that these figures are obtained by comparing GLP-1RA or SGLT-2i with their respective placebos. To date, it is very unlikely that dipeptidyl peptidase-4 inhibitors have a clinically important cardiorenal benefits (Fig. 1): residual risk is 99% for MACE, 100% for kidney outcomes (excluding new albuminuria) [15], and 100% for HF [13]. As CVOT were designed to obtain glycemic equipoise, in order to avoid the interfering influence of different glycemic control, it is still unknown whether the cardiorenal benefits of both intensive glucose lowering and newer drugs are additive. If so, we would expect a further amelioration of cardiorenal outlook in T2D, with the theoretical possibility that the removed risk with SGLT-2i, for example, would surpass 50% for kidney outcomes and 20% for MACE. Although the results of some multinational observational cohort studies suggest this possibility [16, 17], only controlled trials specifically designed to test this possibility would answer the question whether the cardiorenal protection by newer antihyperglycemic drugs is additive to that of optimal glycemic control in T2D.

**Fig. 1:**
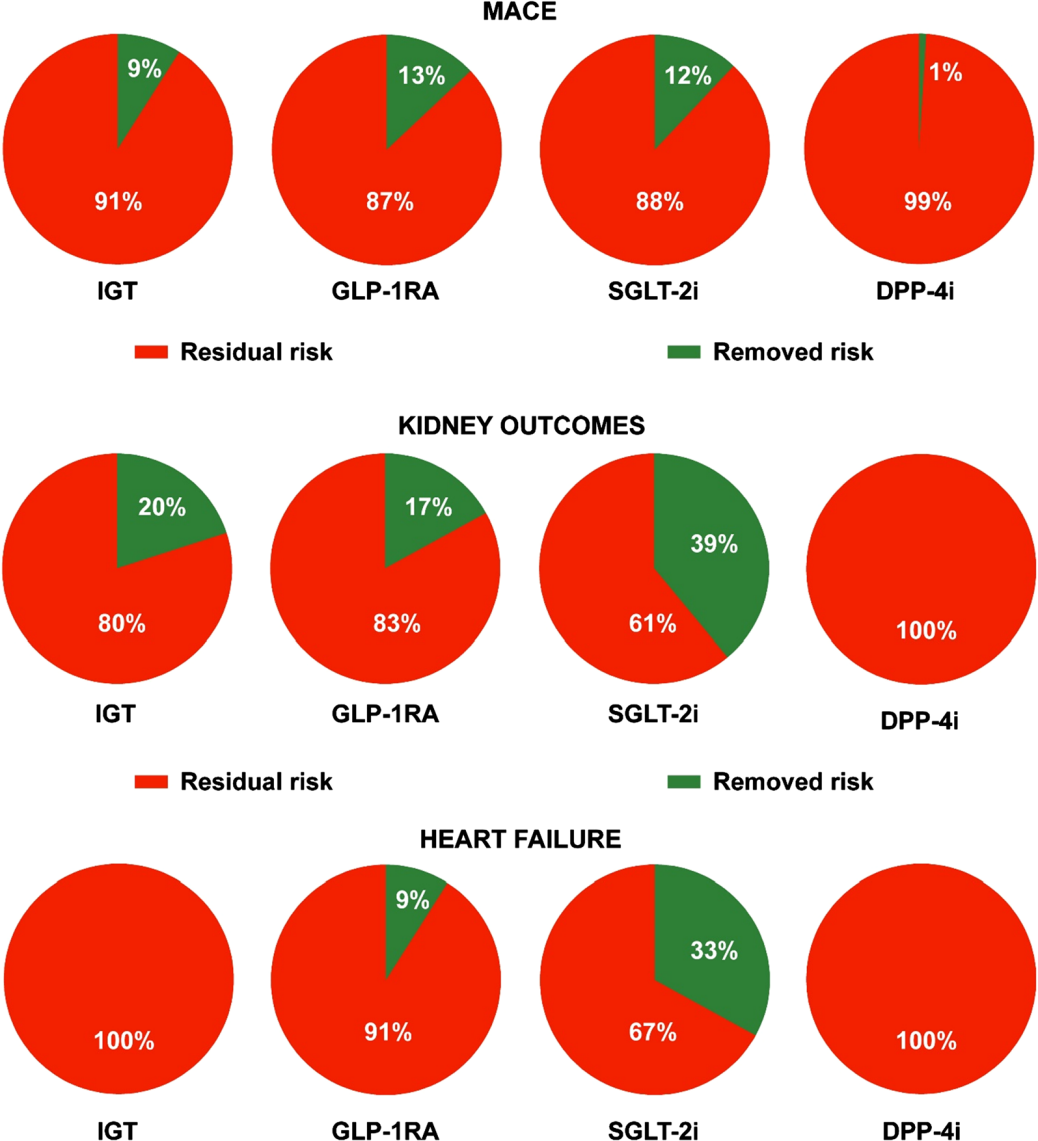
Overview of the residual risk (red) and removed risk (green) of MACE (major adverse cardiovascular events), kidney outcome (in general a composite of sustained decline in the eGRF of at least 50%, end-stage kidney disease, and death from renal cause) and hospitalization from heart failure in intensive glucose trials (IGT) and CVOT (cardiovascular outcome trials) with newer drugs (*GLP-1RA* glucagon-like peptide-1 receptor agonists, *SGLT-2i* sodium-glucose trasporter-2 inhibitors, *DPP-4i* dipeptidyl-peptidase-4 inhibitors) in type 2 diabetes

In conclusion, the results of the many controlled trials (IGT and CVOT) so far concluded have shifted the therapeutic paradigm of T2D, from just, and sometimes obsessive, optimal glycemic control to a more comprehensive cardiorenal protection. In this perspective, and with the awareness that glycemic control may not be enough [18], clinicians are exhorted to prescribe antihyperglycemic drugs with proven cardiorenal benefits [19]. As prescriptions of these agents continue to stagnate, even among eligible patients [20], acknowledging the potential additive role for the blood glucose reduction in decreasing the cardiorenal risk of patients with T2D may overcome some residual reluctance of conservative specialists, who are still not ready to acknowledge that drugs might cut down diabetic cardiorenal complications independently of blood glucose reduction.

## Data Availability

All data generated or analyzed during this study are included in this published article.

## Abbreviations

SGLT-2i: Sodium–glucose transporter-2 inhibitors
GLP-1RA: Glucagon-like peptide-1 receptor agonists
DPP-4i: Dipeptidyl peptidase-4 inhibitors
ESKD: End stage kidney disease
HF: Heart failure
T2D: Type 2 diabetes
CV: Cardiovascular
MACE: Major adverse cardiovascular events
DKD: Diabetic kidney disease
eGFR: Estimated glomerular filtration rate
IGC: Intensive glucose control
IGT: Intensive glucose trials
CONTROL: Collaborators on Trials of Glucose Lowering
UKPDS: UK Diabetes Prospective Study
ACCORD: Action to Control Cardiovascular Risk in Diabetes
ADVANCE: The Action in Diabetes and Vascular Disease: Preterax and Diamicron Modified Release Controlled Evaluation
VADT: Veterans Affairs Diabetes Trial
HbA1c: Hemoglobin A1c
CVD: Cardiovascular disease
HR: Hazard Ratio
CVOT: Cardiovascular outcome trials
